# Risk Analysis of Needle Injury to the Long Thoracic Nerve during Ultrasound-Guided C7 Selective Nerve Root Block

**DOI:** 10.3390/medicina57060635

**Published:** 2021-06-19

**Authors:** Seok Kang, Ha-Mok Jeong, Beom-Suk Kim, Joon-Shik Yoon

**Affiliations:** 1 Department of Rehabilitation Medicine, Korea University Guro Hospital, Seoul 08308, Korea; caprock@paran.com (S.K.); jhmgkahr@naver.com (H.-M.J.); 2 Uijeongbu Eulji Medical Center, Department of Rehabilitation Medicine, Uijeongbu 11759, Korea; mattkim9966@gmail.com

**Keywords:** long thoracic nerve, ultrasound, cervical nerve root, selective nerve root block

## Abstract

*Background and Objectives*: Ultrasound (US)-guided cervical selective nerve root block (SNRB) is a widely used treatment for upper limb radicular pain. The long thoracic nerve (LTN) passes through the middle scalene muscle (MSM) at the C7 level. The needle trajectory of US-guided C7 SNRB pierces the MSM, therefore indicating a high probability of injury to the LTN. We aimed to identify the LTN and to investigate the risk of needle injury to the nerve during US-guided C7 SNRB. *Materials and Methods:* This retrospective observational study included 30 patients who underwent US-guided SNRB at the C7 level in a university hospital. We measured the maximal cross-sectional diameter (MCSD) of the LTN and cross-sectional area (CSA) of the C7 nerve root and assessed the injury risk of LTN during US-guided C7 SNRB by simulating the trajectory of the needle in the ultrasound image. *Results:* The LTN was detectable in all the cases, located inside and outside the MSM in 19 (63.3%) and 11 (36.7%) of cases, respectively. The LTN’s mean MCSD was 2.10 mm (SD 0.13), and the C7 root’s CSA was 10.78 mm^2^ (SD 1.05). The LTN location was within the simulated risk zone in 86.7% (26/30) of cases. *Conclusion:* Our findings suggest a high potential for LTN injury during US-guided C7 SNRB. The clear visualization of LTNs in the US images implies that US guidance may help avoid nerve damage and make the procedure safer. When performing US-guided C7 SNRB, physicians should take into consideration the location of the LTN.

## 1. Introduction

Cervical transforaminal steroid injection (TFSI) is an effective treatment for radiating pain in the upper limb caused by cervical disc disorders [1,2]. TFSI is usually conducted under fluoroscopy or computed tomography (CT) guidance. However, such a procedure bears the risk of accidental intravascular injection and can cause fatal complications, such as brain stem and spinal cord infarctions [3]. In contrast, ultrasound (US)-guided cervical selective nerve root block (SNRB) is a safer alternative procedure because it can be performed while identifying dangerous structures, including blood vessels and peripheral nerves around the target nerve root [4,5,6,7,8].

Progress in high-resolution ultrasound technology has enabled the visualization of small peripheral nerves. In several studies, the brachial plexus and small nerves around the neck were observed using US [9,10,11,12,13,14,15]. The long thoracic nerve (LTN) originates from the anterior branches of the 5th, 6th, and 7th cervical nerve roots and travels to the supraclavicular region after piercing the middle scalene muscle (MSM) [15]. The proximal portion of the LTN is formed by the upper portion stemming from the C5 and C6 nerve roots and the lower portion stemming from the C7 nerve root [16]. In 2015, Lieba-Samal et al. reported that LTN could be reliably visualized using ultrasound [11]. The LTN appeared as a hyperechoic fascial line running within the MSM on the US image. The branches from the C5 and C6 nerve roots converged to the main LTN within this fascia.

Two studies have investigated the dorsal scapular nerve (DSN) and LTN during US-guided interscalene brachial plexus block using a posterior approach [10,12]. In both studies, the authors identified nerves within the MSM. According to Kim et al., there could be a risk of injury to the DSN and LTN during the procedure [12].

When performing US-guided cervical SNRB, the transducer is applied transversely to the lateral aspect of the neck. The needle is inserted on the posterolateral side and advances obliquely toward the target nerve root. The trajectory should pass through the MSM during the procedure at the C7 level. Since the superior portion of the LTN is usually found within the MSM, it may be in the middle of the needle’s trajectory. We thought that needle injury is more likely to occur in the LTN during US-guided C7 SNRB. This study aimed to identify the LTN around the C7 transverse process in an ultrasound image and investigate the possibility of LTN injury during US-guided C7 SNRB. We hypothesized that an area between the needle’s paths during the procedure is the area with a high risk of injury. We evaluated whether the LTN was inside the risk area on the US image.

## 2. Materials and Methods

This retrospective observational study was approved by the institutional review board of our hospital (2020GR0355). We reviewed and analyzed the US images of patients who underwent US examination or US-guided SNRB at the C7 level from September 2017 to March 2018.

We included patients with the following criteria in this study:

(1) Age 18 years or older;
(2) Patients who underwent US examination of the C7 nerve root and LTN;
(3) Patients in whom the evaluation of the LTN was performed, among those who underwent US-guided C7 SNRB.

Those who met the following criteria were excluded:

(1) Patients who underwent surgery on the 7th cervical spine;
(2) Patients with systemic diseases that could cause peripheral nerve abnormalities, including diabetes or chronic kidney disease;
(3) Patients confirmed with LTN abnormalities due to trauma or other causes.

### 2.1. Ultrasound Image

US examination was performed using a 12-MHz linear transducer of a US machine (Philips Ultrasound Inc., Bothell, WA, USA). We examined patients with the head turned to the opposite side in the supine position. A single examiner with more than 10 years of experience in peripheral nerve ultrasound performed all examinations. The cervical nerve root level was confirmed by the characteristic shape of the transverse process of the vertebrae. We identified the C5 transverse process by the “two-humped camel” sign formed by the anterior and posterior tubercles. The C6 showed a prominent anterior tubercle, and C7 had a rudimentary anterior tubercle and prominent posterior tubercle. Tracing the nerve roots from the C5 to C7 level, we identified the branches from the C5 and C6 nerve roots merging into the LTN around the MSM. The LTN, C7 nerve root, and posterior tubercle of the C7 transverse process were captured on a single image. We measured the maximal cross-sectional diameter (MCSD) of the LTN rather than the cross-sectional area (CSA) because a high measurement error is expected for the CSA of very small nerves such as the LTN. We also measured the maximal diameter, minimal diameter, and CSA of the C7 nerve root and analyzed the positional relationship between each structure captured on the US image. The diameters and CSA were measured three times to minimize measurement errors, and the average values were recorded.

### 2.2. Simulated Risk Analysis of Long Thoracic Nerve Injury

**Figure 1:**
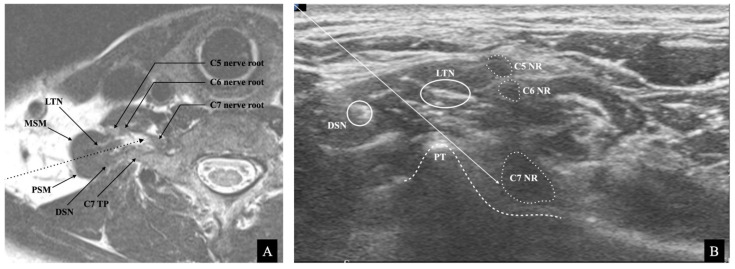
The needle’s trajectory and anatomical relationship of the long thoracic nerve (LTN) and dorsal scapular nerve (DSN) during the ultrasound-guided C7 selective nerve root block (SNRB). (**A**) A T2-weighted magnetic resonance image around the C7 nerve root. The needle’s trajectory (dotted arrow) during the C7 SNRB pierces the middle scalene muscle (MSM). The LTN is near the trajectory within the MSM. The dorsal scapular nerve (DSN) around the posterior scalene muscle (PSM) is away from the trajectory. (**B**) The ultrasound image of the C7 SNRB. The needle’s trajectory (arrow) goes over the posterior tubercle (PT) of the C7 transverse process and reaches the C7 nerve root (NR). The LTN is located near the trajectory, and the DSN is behind the PT away from the trajectory.

**Figure 2:**
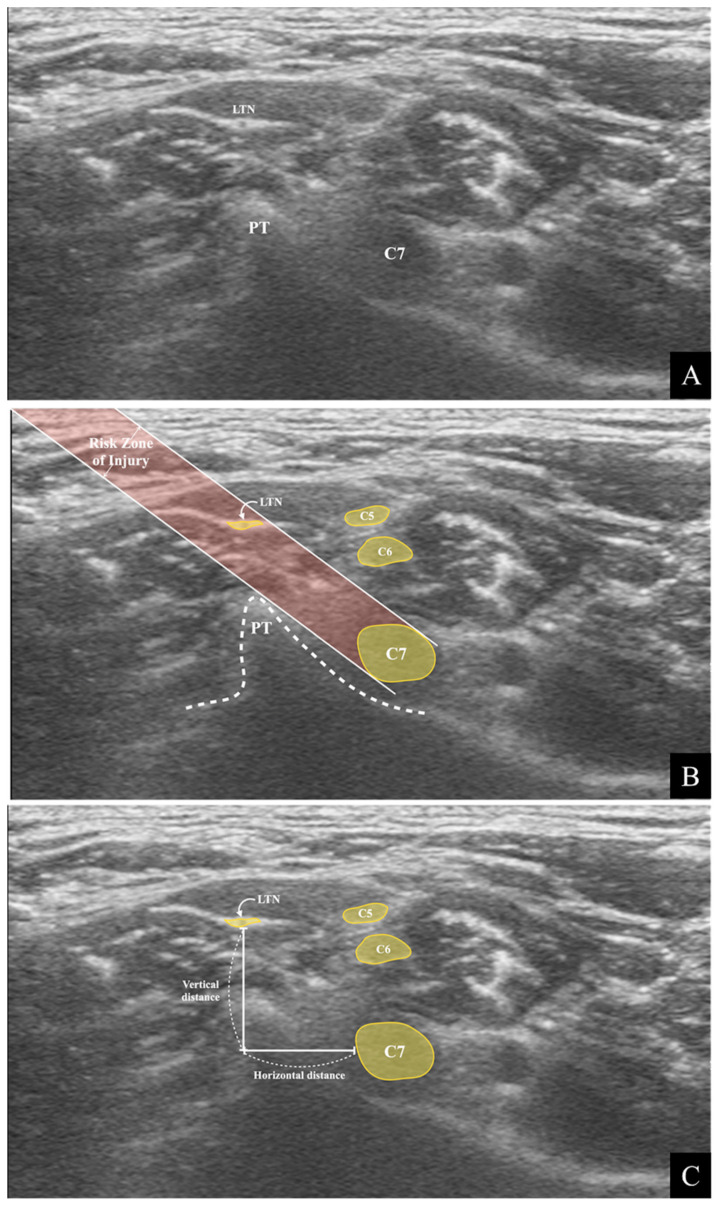
The analysis and measurement of the ultrasound image. (**A**) The ultrasound image captures the C7 transverse process’s posterior tubercle (PT), C7 nerve root, and long thoracic nerve (LTN). (**B**) The simulated analysis of the injury risk of the LTN. Two imaginary lines are drawn, and the area between the lines is the risk zone of injury. (**C**) The distance measurement of the LTN from the C7 nerve root.

During the US-guided C7 SNRB, the trajectory of the needle is oblique from the posterolateral side of the neck to the posteroinferior side of the C7 nerve root. The needle should pierce the MSM and pass over the posterior tubercle of the C7 transverse process (Figure 1). We drew two imaginary lines simulating the trajectory of the needle on the ultrasound image to assess the risk of needle injury to the LTN within the MSM during US-guided C7 SNRB (Figure 2B). The two lines were drawn as follows:

(1) A straight oblique line starting from the skin, passing through the tip of the posterior tubercle, and reaching the posteroinferior rim of the C7 nerve root.
(2) A line parallel to line (1), starting from the skin and connecting to the superior margin of the C7 nerve root.

The hypothetical risk zone of needle injury was assumed to be the area between the imaginary lines. The LTN location related to the risk of injury area was investigated. We also measured the vertical and horizontal distances of the LTN from the C7 nerve root (Figure 2C).

### 2.3. Statistical Analysis

The sample size was calculated based on the results of a previous study about ultrasound identification of the LTN during interscalene block [10]. G*Power 3.1.9 software was used to calculate the sample size of the binomial test, one sample case. When the effect size was 0.23, α was 0.05, and (1 − β) was 0.8, and the required total sample size was 30.

We used SPSS version 22 to calculate all statistical values. The obtained data were tested for normality using the Shapiro–Wilk test (Supplementary File). According to the normality test, all the data were normally distributed except the age of the patients. The mean values and standard deviations were calculated for the data in the normal distribution, and the median value and interquartile range were obtained for the age. We used the Mann–Whitney test to compare the horizontal and vertical distances of the LTN from the C7 nerve root between the groups of the LTN inside and outside the risk area. Although the values were normally distributed, the nonparametric test was used because the sample size of the group with the LTN outside the risk zone was extremely small. We used the chi-square test to analyze the LTN location related to the MSM and the simulated risk zone.

## 3. Results

Twenty patients were enrolled in this study. A total of 30 cases were assessed because 10 patients underwent US examination on both sides. The demographic characteristics are presented in Table 1.

**Figure 3:**
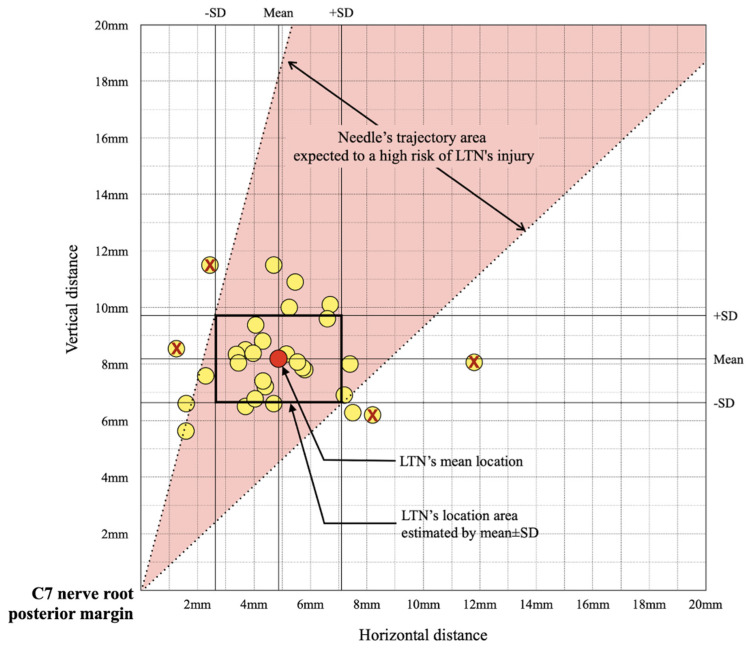
The schematic diagram reveals the long thoracic nerve (LTN)‘s location of each case. The C7 nerve root’s posterior margin is the origin, the *x*-axis is the horizontal distance, and the *y*-axis is the vertical distance. The LTN marked as 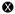 indicates that it was outside the risk area in the simulated risk analysis. The LTN’s mean location and locational area estimated by the mean ± standard deviation (SD) are presented. The painted area indicates that the needle’s trajectory is expected to be associated with a high risk of LTN injury.

We distinctly detected the LTN in all cases. The LTN’s location at the C7 transverse process level was inside the MSM in 19 cases (63.3%) and outside in 11 cases (36.7%). In analyzing the injury risk in US-guided C7 SNRB, we found that 86.7% of cases had an LTN inside the risk area simulating the needle’s path. Table 2 lists the measurement data for each case. The mean MCSD of LTN was 2.10 mm (SD 0.13). The mean maximal diameter, minimal diameter, and CSA of the C7 nerve root were 4.00 mm (SD 0.39), 3.39 mm (SD 0.25), and 10.78 mm^2^ (SD 1.05), respectively. The mean distance of the LTN’s location from the C7 nerve root horizontally was 4.87 mm (SD 2.23) and vertically was 8.18 mm (SD 1.54). In Figure 3, we represent the LTN locations of each case from the C7 nerve root of the starting point using the horizontal and vertical distance data as coordinates. Furthermore, we indicate the LTN’s mean location and the location range calculated using the standard deviation. The needle’s trajectory area, which has a high probability of encountering the LTN on average when approaching the C7 nerve root, was painted.

Table 3 shows the results of the statistical analyses. In cases where the LTN was outside the risk zone, the horizontal distance between the LTN and C7 nerve root was not significantly different (*p* > 0.05). There was a slight difference in the vertical distance between the cases with the LTN inside and outside the risk zone, but the statistical significance was insufficient (*p* = 0.063). There was no significant correlation between the LTN location and injury risk.

## 4. Discussion

In this study, we aimed to investigate the possibility of LTN injury during US-guided C7 SNRB. The result stating that the LTNs were within the risk zone in 86.7% of cases, which suggests a high risk of nerve injury during the procedure. In addition, this study’s findings imply that US guidance could help avoid the risk because LTNs were easily detectable using US in all cases. This is the first study to identify the risk of LTN damage during US-guided cervical SNRB.

Several researchers have investigated the US anatomy of the LTN at the cervical spine level. Chang et al. described the sono anatomy and techniques for observing the LTN [15]. They reported that the proximal portion of LTNs might be challenging to visualize because of their small diameter and complicated branching pattern. In addition, investigators could misrecognize the DSN arising from the C5 nerve root [15]. In this study, we cautiously detected the LTN and distinguished it from the DSN by tracing the branches between the C5 and C7 nerve roots. The DSN traveled posterior to the LTN at the C7 transverse process level and was found behind the posterior tubercle away from the trajectory of the C7 SNRB (Figure 1). Thus, we did not include the DSN for measuring and analyzing the US images in this study.

Lieba-Samal et al. reported that LTNs were clearly observed within the MSM in all cases. In their study, the LTN mean MCSD was 1.6 mm (SD 0.3) [11]. The MCSD also has been reported in the other anatomical studies at 2.27 mm (SD 0.39) in Wang et al.’s study [17] and 3 mm (SD 2.5) in Tubbs et al.’s study [18]. In our study, the LTNs were detectable in all cases as well, and the result of the MCSD (2.10 mm, SD 0.13) was not significantly different from the previous reports. However, since we included only the superior trunk of the LTN that originated from the C5–6 nerve roots in the measurement of the MCSD, there might be some discrepancies. In addition, the studies of Wang et al. and Tubbs et al. were cadaver dissection studies; therefore, the results of ultrasonographic measurements in this study cannot be directly compared. Nevertheless, it was reported that the diameter of the superior trunk of the LTN was approximately 2 mm (range 1.5–2.8 mm) in Tubbs et al.’s study [18], which is similar to our results.

Multiple studies have suggested that the superior portion (from C5–6 roots) and the inferior portion (from C7 root) of the LTN join to form the main branch [16,17,18,19]. However, diverse variations in the cervical nerve roots contribute to LTN. In several studies, LTN was reported to occasionally originate from the C4 or C8 nerve root [17,19,20]. In an ultrasound observational study by Lieba-Samal et al., the C7 nerve root contribution was found in only 28% of volunteers [11]. Yazar et al. suggested four types of LTN after dissection of 21 cases of 12 embalmed cadavers [19]. In that study, one type (type 3, 14.3% of cases) showed that the C7 nerve root did not contribute to the LTN, and there were two types (type 2, 33.3%; and type 4, 4.8%) in which the C5 nerve root did not give a branch. However, in all types of LTNs, the C6 nerve root contributed to the nerve. In the present study, the C5 and C6 nerve roots distinctly appeared to form the superior portion of the LTN in all cases; however, it required cautious tracing to detect the branch from the C5 root because it was difficult to differentiate it from the DSN. This study aimed to investigate the ultrasonographic features and anatomical relationships of the LTN in US-guided C7 SNRB; therefore, we did not consider a branch from the C7 nerve root for investigation.

**Figure 4:**
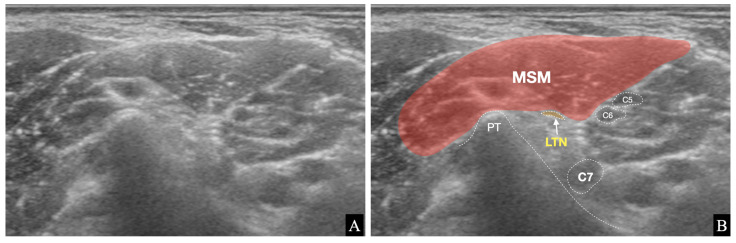
A case that the long thoracic nerve (LTN)’s location is outside of the middle scalene muscle (MSM). (**A**) A pure ultrasound image without indications of structures. (**B**) The posterior tubercle (PT), MSM, LTN, and C5–7 nerve roots are indicated on the ultrasound image.

In anatomical studies, the positional relationship between the LTN and MSM has been investigated. Yazar et al. found that C5–6 components of the LTN were between the middle and posterior scalene muscles in 47.6% of 21 cases, pierced the MSM in 33.3% of cases, and coursed over the MSM in 19% of cases [19]. In the study by Tubbs et al., the superior component from C5–6 nerve roots of the LTN was between the MSM and posterior scalene muscle in 56% of cases and traveled through the MSM in 11% of cases [18]. In contrast, in ultrasonographic observational studies, LTNs have been found within the MSM [10,11,12]. In the present study, we found the LTN within the MSM in most cases (63.3%). In 11 cases (36.7%), however, the LTN was located outside the MSM without piercing (Figure 4).

**Figure 5:**
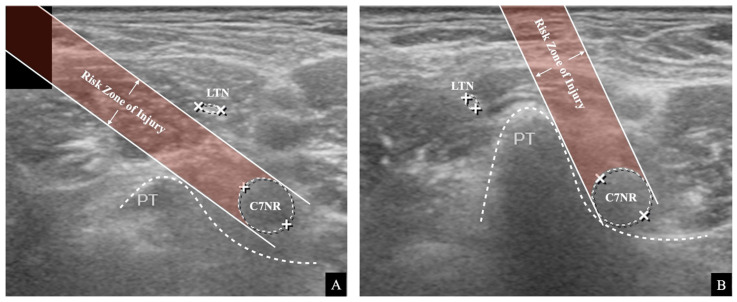
Cases in which the long thoracic nerve (LTN)’s location is outside of the risk zone. (**A**) The LTN is anterior to the risk zone. The C7 transverse process’s posterior tubercle (PT) shape is shallow, so the simulated needle’s trajectory line is relatively horizontal. (**B**) The LTN is posterior to the risk zone. The needle’s trajectory line is relatively vertical due to the prominent PT, and the LTN travels behind the PT.

There have been two studies on the risk of encountering the DSN and LTN during the US-guided interscalene brachial plexus block (ISBPB) [10,12]. In both studies, the DSN was more likely to be encountered than the LTN. Hanson and Auyong reported that they identified DSN and LTN in 77% and 23% of 50 cases, respectively [10]. In Kim et al.’s study, the DSN, LTN, both nerves, and neither nerves were encountered in 44 (62.8%), 15 (21.4%), 10 (14.3%), and 21 (30.4%) cases, respectively [12]. In the US-guided ISBPB, the probe moved more distally than the C7 SNRB. Furthermore, the needle insertion point was more posterior, and the trajectory was more horizontal in the US image. Therefore, DSNs located posterior to the LTNs would have a high probability of encountering the path of the needle when performing US-guided ISBPB. On the other hand, when performing a US-guided C7 SNRB, the needle trajectory is more vertical and the location of the DSN is away from the needle’s path. Thus, we focused on the fact that LTN is more likely to be injured during the procedure. In this study, LTN was found within the risk zone in 86.7% of the cases. In cases outside the simulated risk zone, the LTN could be found anterior or posterior to the risk zone. The injury risk from the needle approach during C7 SNRB could depend on the locational relationship between the LTN and the posterior margin of the C7 nerve root. Thus, we estimated the needle path area with a high risk of injury (Figure 3). However, there was no significant relationship between location data and the risk of injury. The LTN location related to the MSM was also not significantly associated with the risk of injury (Table 3). Instead, the posterior tubercle’s shape seems to influence the risk analysis result because it determines the simulated trajectory lines (Figure 5). In future studies, it would be necessary to investigate the relationship between the shape of the posterior tubercle and injury risk.

**Figure 6:**
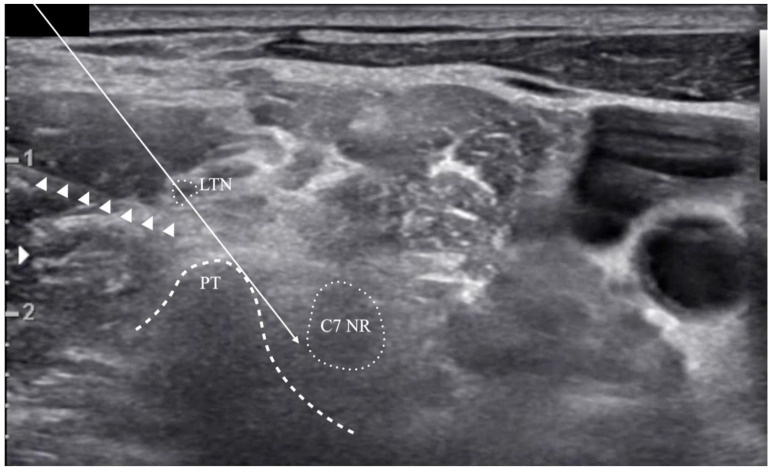
A captured ultrasound image of the C7 selective nerve root block. The arrow is the simulated needle’s trajectory. The long thoracic nerve (LTN) is in the middle of the trajectory; thus, the needle’s direction (arrowheads) should be modified. PT, posterior tubercle; NR, nerve root.

US-guided cervical SNRB is a useful management tool for upper limb radiating pain. It is well known that US-guided procedures have the advantage of being safer than fluoroscopy-guided procedures. Many physicians consider the risk of intravascular injection while performing US-guided cervical SNRB. Recent advances in ultrasound technology have enabled the observation of small soft-tissue structures. Thus, we have noticed that small nerves, such as the LTN, are at risk of injury during US-guided cervical SNRB. This study evaluated the risk by simulating the needle trajectory when performing C7 SNRB. There might be a limitation in that we did not confirm the LTN by inserting the needle and stimulating it. However, the result of 86.7% of cases having the risk suggests that the LTN should be considered when determining the needle’s trajectory. If the LTN is found within the needle path, physicians should modify the direction of the needle (Figure 6). In addition, using an electrostimulation needle would be suggested to avoid accidental LTN injury if it is difficult to locate the nerve during the procedure.

This study had some limitations. First, this was a retrospective observational study. There was no control over variables that could affect the ultrasound findings of peripheral nerves, such as the age, sex, and body mass index of the subjects. Second, the number of participants was small. To avoid bias and control confounding factors, a prospectively designed study with a large number of subjects would be necessary for further investigation. Third, a single researcher conducted the US investigation. Although an experienced examiner performed the US using a verified method, the results of one examiner may be inaccurate compared to the results obtained by consensus among multiple examiners. In the future, it will be necessary to analyze the results that are consistent with several blinded examiners after conducting a standardized method.

## 5. Conclusions

Our findings indicate the potential for LTN injury during US-guided C7 SNRB. More importantly, the clear visualization of LTNs in US images of the procedure suggests that US guidance may help avoid nerve damage and make the procedure safer. When performing US-guided C7 SNRB, physicians should pay attention to the location of the LTN.

## Figures and Tables

**Table 1:** Demographic characteristics of the subjects.

Numbers of patients	20
Numbers of cases	30
Age (years)	35.5 (30–59.75)
Sex (male:female)	14:6
Height (cm)	170.00 ± 8.10
Weight (kg)	65.45 ± 10.92
Side (right:left)	17:13

The values for height and weight are presented as mean ± standard deviation. The age is presented as median (interquartile range).

**Table 2:** Measurement data of the long thoracic nerve (LTN) and C7 nerve root.

Case No.	LTN	C7 Nerve Root			Horizontal Distance (mm)	Vertical Distance (mm)
	MCSD (mm)	Maximal Diameter (mm)	Minimal Diameter (mm)	CSA (mm^2^)		
1	2.04	3.70	3.18	9.52	6.7	10.1
2	2.18	3.83	3.34	10.2	6.6	9.6
3	2.23	4.65	3.09	11.53	5.8	7.8
4	1.93	4.13	3.15	10.46	8.2	6.2
5	1.93	3.65	3.36	9.76	3.7	8.5
6	1.87	3.64	3.03	8.72	3.7	6.5
7	2.23	4.81	3.08	11.84	7.2	6.9
8	2.23	3.86	3.70	11.29	4.7	6.6
9	2.14	3.75	3.67	10.99	4.4	7.2
10	2.09	3.71	3.38	9.97	7.4	8
11	2.09	3.99	3.32	10.62	11.8	8.06
12	1.98	3.58	3.53	10.09	7.5	6.28
13	2.23	4.03	3.89	12.36	3.38	8.34
14	1.98	3.60	3.59	10.23	5.25	10
15	2.23	3.74	3.50	10.44	5.15	8.35
16	2.31	3.88	3.72	11.63	5.72	7.87
17	1.98	3.96	3.15	9.99	4.06	9.38
18	1.98	3.76	3.50	10.46	2.44	11.5
19	2.14	3.97	3.66	11.51	2.29	7.58
20	2.09	3.49	3.35	9.44	4.32	7.4
21	2.04	3.85	3.28	10.13	5.46	10.9
22	2.23	3.84	3.79	11.7	3.45	8.04
23	2.23	4.28	3.27	11.24	4.04	6.77
24	2.31	4.26	3.81	12.87	5.54	8.07
25	2.04	3.68	3.24	9.38	4.31	8.81
26	1.98	4.65	3.10	11.56	1.59	5.63
27	2.31	5.14	3.20	13.08	1.6	6.6
28	2.04	4.33	3.41	11.77	1.25	8.54
29	2.04	4.12	3.10	10.2	3.97	8.38
30	1.93	3.98	3.29	10.49	4.7	11.5

MCSD, maximal cross-sectional diameter; CSA, cross-sectional area.

**Table 3:** The statistical analysis of the association between the long thoracic nerve’s locational relation to the C7 nerve root and middle scalene muscle and injury risk.

	LTN’s Location		*p*-Value
	Inside the Risk Zone (*n* = 26)	Outside the Risk Zone (*n* = 4)	
Horizontal distance (mm)	4.55 (3.70–5.74)	4.52 (1.55–10.50)	0.903
Vertical distance (mm)	7.94 (6.73–8.58)	9.07 (8.18–11.03)	0.063
LTN inside the MSM (%)	65.4	50	0.552

LTN, long thoracic nerve; MSM, middle scalene muscle. The values for the distances are presented as median (interquartile range). The p-value was calculated using the Mann–Whitney test for the horizontal and vertical distance analysis and the chi-square test for the LTN’s location for the MSM.

## Data Availability

The data presented in this study are available on request to the corresponding author.

## Supplementary Materials

The following are available online at https://www.mdpi.com/article/10.3390/medicina57060635/s1, Table S1: The results of the normality test, Table S2: The normality test results in groups classified according to the risk of injury to the LTN.
