# Supplementary material for: Risk Analysis of Needle Injury to the Long Thoracic Nerve during Ultrasound-Guided C7 Selective Nerve Root Block
Source: Medicina (Kaunas). 2021 Jun 19;57(6):635. doi: 10.3390/medicina57060635 (PMC8235341; doi:10.3390/medicina57060635)
Supplement: Supplementary file 1 [file medicina-57-00635-s001.zip › Supplementary file.pdf]

Table S1. The results of the normality test.

Variable	N	*p*-Value
Age	20	0.004
Height	20	0.517
Weight	20	0.104
MCSD of LTN	30	0.050
CSA of C7	30	0.540
Horizontal distance	30	0.136
Vertical distance	30	0.170

p-values were calculated by the Shapiro-Wilk test.

Table S2. The normality test results in groups classified according to the risk of injury to the LTN.

	LTN Inside the Risk Zone		LTN Outside the Risk Zone	
	N	*p*-Value	N	*p*-Value
Horizontal distance	26	0.608	4	0.556
Vertical distance	26	0.250	4	0.561

p-values were calculated by the Shapiro-Wilk test.
